# Osteoblast and stem cell response to nanoscale topographies: a review

**DOI:** 10.1080/14686996.2016.1242999

**Published:** 2016-11-04

**Authors:** Nur Izzati Aminuddin, Roslina Ahmad, Sheikh Ali Akbar, Belinda Pingguan-Murphy

**Affiliations:** ^a^ Department of Biomedical Engineering, Faculty of Engineering, University of Malaya, Kuala Lumpur, Malaysia; ^b^ Department of Mechanical Engineering, Faculty of Engineering, University of Malaya, Kuala Lumpur, Malaysia; ^c^ Department of Materials Science and Engineering, The Ohio State University, Columbus, OH, USA

**Keywords:** Cellular response, nanotopography, rare earth metal oxides, self-assembly, stem cell, 30 Bio-inspired and biomedical materials, 102 Porous / Nanoporous / Nanostructured materials, 211 Scaffold / Tissue engineering/Drug delivery, 212 Surface and interfaces

## Abstract

To understand how cells respond to the nanoscale extracellular environment *in vivo*, cells from various sources have been cultured on nanoscale patterns fabricated using bottom-up and top-down techniques. Human fetal osteoblasts (hFOBs) and stem cells are some of them and they are known to be overtly responsive to nanoscale topographies – allowing us to investigate the hows and whys of the response *in vitro*. Information gathered from these *in vitro* studies could be used to control the cells, i.e. make the stem cells differentiate or retain their characteristics without the use of medium supplements. In this review, hFOB and stem cell responses to nanotopographies are summarized and discussed to shed some light on the influence of patterns on the reactions. Although both types of cells are responsive to nanoscale topographies, the responses are found to be unique to topographical dimension, shape, orientation and the types of cells used. This implies that cellular responses are influenced by multitude of factors and that if done right, cheaper self-assembled nanotopographies can be tailored to control the cells. A new self-assembly, powder-based technique is also included to provide an insight into the future of nanofabrication.

## Graphical abstract

**Figure:**
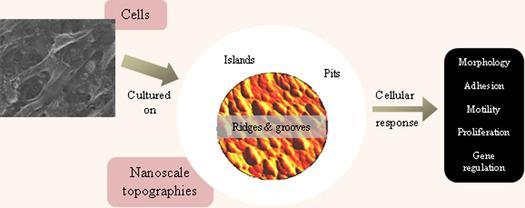

## 1. Introduction

Cells in living organisms are surrounded by and in contact with nanoscale topographic interfaces. These interfaces, commonly known as basement membranes, are composed of a complex mixture of pits, pores, protrusions, ridges and fibers, with sizes between 5 and 200 nm.[1] Basement membrane is a component of extracellular matrix (ECM) and plays an important role in tissue development and organization. It separates endothelia, epithelia, muscle fibers, and nervous systems from the connective tissue.[2]

Although cells are known to interact with their surroundings *in vivo*, the nature of their interactions was not physically observed until Harrison cultured embryonic cells on a spider web. He found that the cells followed the direction of the fibers, which indicates that cells will adjust themselves on the substratal structure.[3] However, in spite of this breakthrough finding, scientists only started to incorporate nanoscale structures in tissue engineering materials after micro- and nanofabrication techniques were widely known and available, just over a decade ago. Studies on these structures have found that they are capable of modulating cellular responses including cell morphology,[4] adhesion,[5] motility,[6] proliferation,[7,8] endocytotic activity,[9] protein abundance,[10,11] and gene regulation.[12,13]

It is imperative to consider topographical cues deliberately or inadvertently presented to cells, as feature size as small as 10 nm has been found to affect cell behavior.[14] This review focuses on osteoblast and mesenchymal stem cell (MSC) response to nanotopographies embossed against polymeric materials. Apparently both types of cells are convenient models for studying cell-topography interaction *in vitro* [15–19] as they are overtly responsive to gross topography of materials. Production of patient-specific tissues with reduced risk of immune rejection [20] or tissues with decreased immunogenicity [21] and potentially immunosuppressive properties [22] for allogenic transplantation [23] by modulating stem cell response using nanotopographies, may solve some of the problems faced in regenerative medicine.

## 2. Osteoblast and stem cell response to nanotopograph

It is widely acknowledged that cells do not interact directly with the surface of a material but with the adsorbed proteins from the ECM.[24,25] In general, when a material is exposed to a biological fluid, such as blood, lymphatic fluid or cell culture medium, water molecules will bind rapidly to the surface and establish a water mono- or bi-layer.[26] Surfaces that have a strong tendency for binding water are called hydrophilic while the opposite are called hydrophobic.[26] After the formation of adsorbed water layer, chlorine ions (Cl^–^) and sodium ions (Na^+^) get incorporated to the surface in which the arrangement is highly influenced by the properties of the surface.[26] Subsequently, proteins from the serum-containing culture medium or synthesized (and secreted) by the cells adsorb to the surface. Smaller proteins are adsorbed to the surface first, followed by conformational changes before finally replaced by larger proteins.[26]

The resulting mixture of proteins on the surface, their conformational state and orientation vary depending on the properties of the surface [27] and very much influence the compliance of the surface for subsequent cell attachment and spreading.[26] Cell attachment and spreading are initiated by specific and nonspecific interactions [26] between the cells and the adsorbed proteins. Nonspecific interactions involve electrostatic, electrodynamic, steric and entropic interactions while specific interactions involve cell-surface receptors called integrins. Basically specific interactions between cell-surface receptors and the adsorbed proteins provide mechanically stable substrate anchorage of the cells.[28,29]

Integrins are the most prominent type of transmembrane receptors that are involved in cell adhesion.[30] They are a family of *α*, *β*-heterodimeric glycoproteins that project 20 nm from the cell membrane [31] and the *β*-subunit on the intracellular side is linked to the actin cytoskeleton [32] by cytoskeletal adapter proteins such as talin, vinculin, *α*-actinin and paxillin.

After ligand binding, integrins will cluster locally and form focal adhesions/contacts.[24,33] Through focal adhesions, cells react to extrinsic chemical and mechanical signals from the cell–cell contact or cell–ECM components.[34] These mechanical signals (forces) will be translated to biochemical signals by the proteins joining the focal adhesion complex before they are passed to the nucleus.[35]

Signal propagation/transmission can be achieved either via direct or indirect mechanotransduction.[34,36] In direct mechanotransduction, information about the ECM topography is passed to the nucleus as changes in focal adhesions and cytoskeletal conformation.[37] In response to tension, the intermediate filaments of the cytoskeleton reorient and cause a distortion in the nucleus which results in nucleoli shifting along the appropriate axis.[38]

Generally, indirect mechanotransduction is achieved through the activation of the extracellular-signal-regulated kinase (ERK)/mitogen-activated protein kinase (MAPK) pathway.[34] MAPK pathway consists of a chain of proteins and is involved in signal propagation from focal adhesion sites to the nucleus,[39] cellular differentiation and cell cycle regulation. Surface topography has been identified as an influential factor in ERK/MAPK signaling [40] through modulation of integrin clustering and adhesion formation.

The size, symmetry (isotropic and anisotropic), and regularity of surface topography have been found to influence cell attachment which leads to adhesion that consequently dictate the alignment, migration, proliferation and differentiation/fate of the cells.[41]

**Figure 1.**
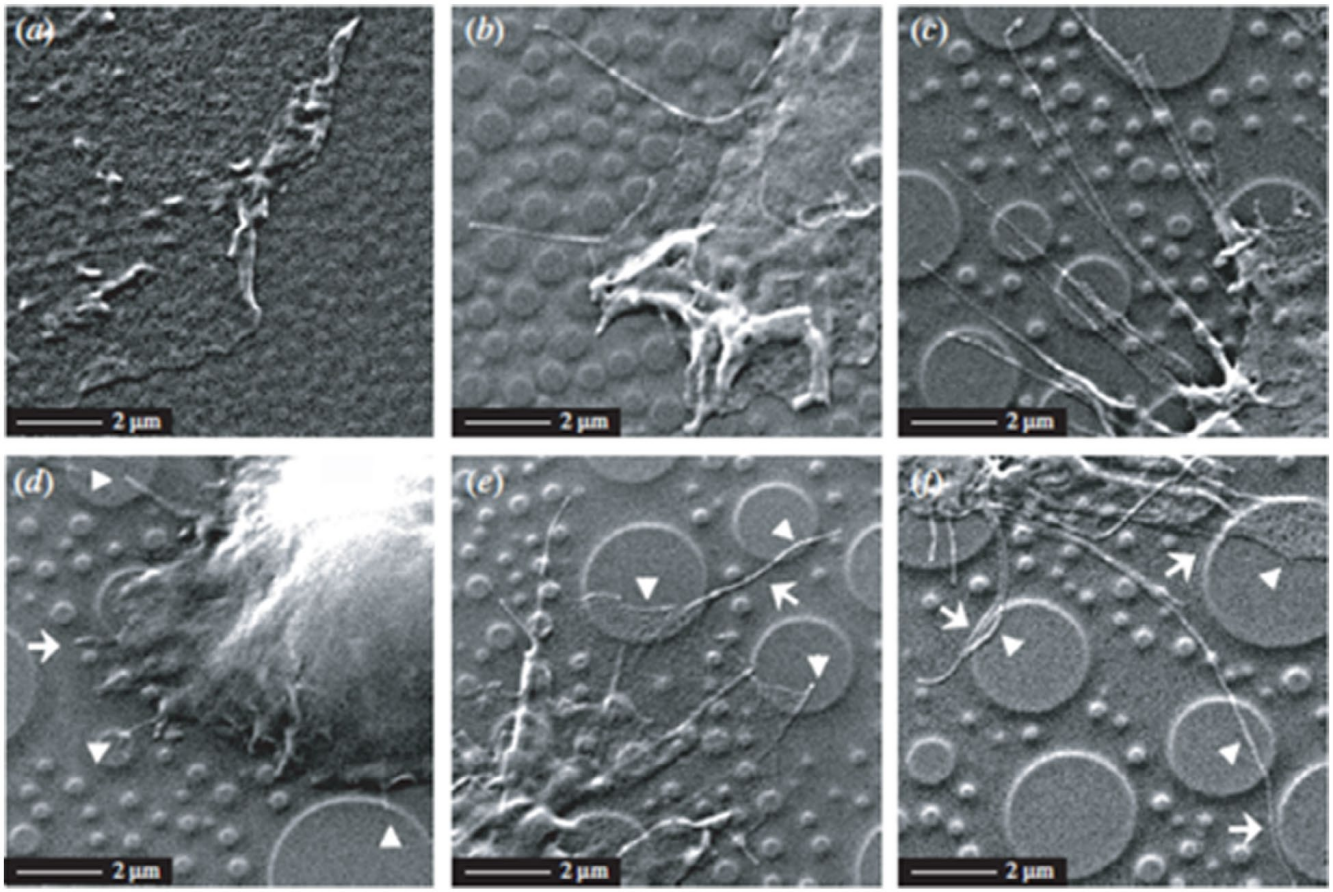
Scanning electron microscope (SEM) images of hFOB cells on (a) 11 nm, (b) 38 nm, and (c) 85 nm islands after cultured for 24 h. Additional SEM images of hFOB cells cultured on 85 nm islands after (d, e) 3 h and (f) 24 h. Arrowhead and arrow indicate the interaction with the top and other portions of island respectively. Images reproduced with permission from [42].

In the study of hFOB cell response to polymer demixed nanotopographic interfaces, randomly distributed nanoisland topography was made by spin casting polystyrene (PS)/polybromostyrene (PBrS) polymer solutions with total polymer concentration of 0.5, 2 and 5% (w/w) to produce nanoscale islands with an average height of 11, 38 and 85 nm respectively (Figure 1).[42] A significant increase in hFOBs was observed on 11 nm nanoislands compared to the flat control after 3 h of culture. Larger cells were also seen on 11 nm nanoislands relative to larger nanoislands and tissue culture polystyrene (TCPS). The author also found no correlation between surface wettability and cell adhesion as cells adhered similarly to surface with different contact angle (38 nm versus PS) and differently to surface with similar contact angles (85 nm versus PS). The trend of decreasing cellular adhesion with increasing nanoprotrusion height is in agreement with findings by Sjostrom et al., who identified reduction in adhesion formation with increasing nanopillars height.[43]

In the focal adhesion model proposed by Lamers et al., osteoblast adhesion is firm and not disrupted on the smooth surface [44] – leading to an assumption of uninterrupted subsequent cell responses on smooth TCPS. What is interesting is that, despite the suppressed proliferation, alkaline phosphatase (ALP) activity on 85 nm nanoislands was comparable to that of smooth TCPS. The review of Biggs et al. [45] mentioned that the mechanisms responsible for the focal adhesion formation in adherent cells could be based on an interplay between two promoting and perturbing mechanisms. Despite being >70 nm in height, the inter-feature spacing (not stated in the study) could have increased the number of available integrin binding sites, leading to similar ALP activity on the 85 nm nanoislands with the TCPS.

**Figure 2.**
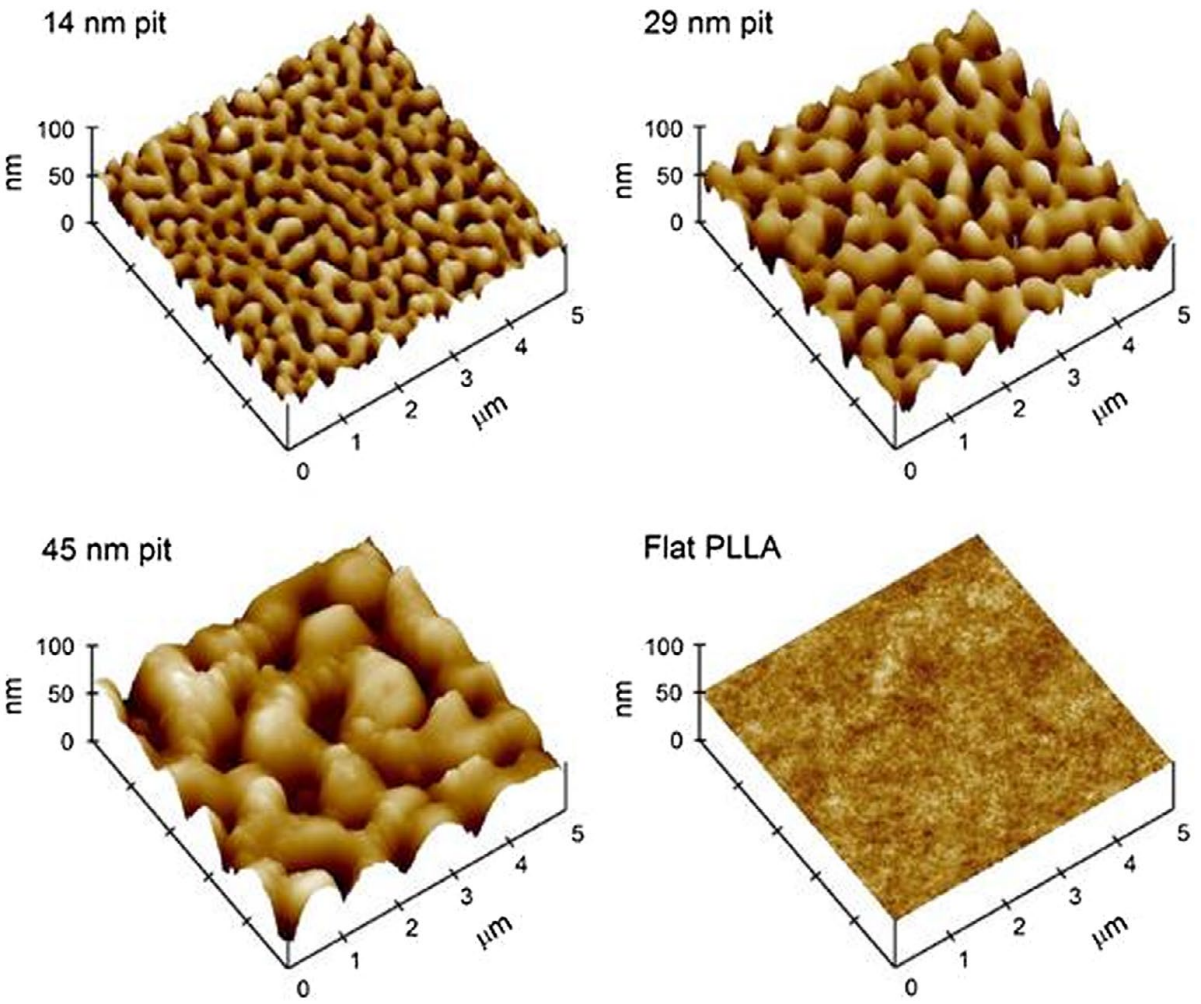
Atomic force microscopy (AFM) images of poly-lactic acid (PLLA)/PS demixed nanopit-textured films spin-cast at 0.5% solution concentration (forming 14 nm deep pits),1% solution concentration (forming 29 nm deep pits), and 1.5% solution concentration (forming 45 nm deep pits) and flat PLLA films. Images reproduced with permission from [46].

**Figure 3.**
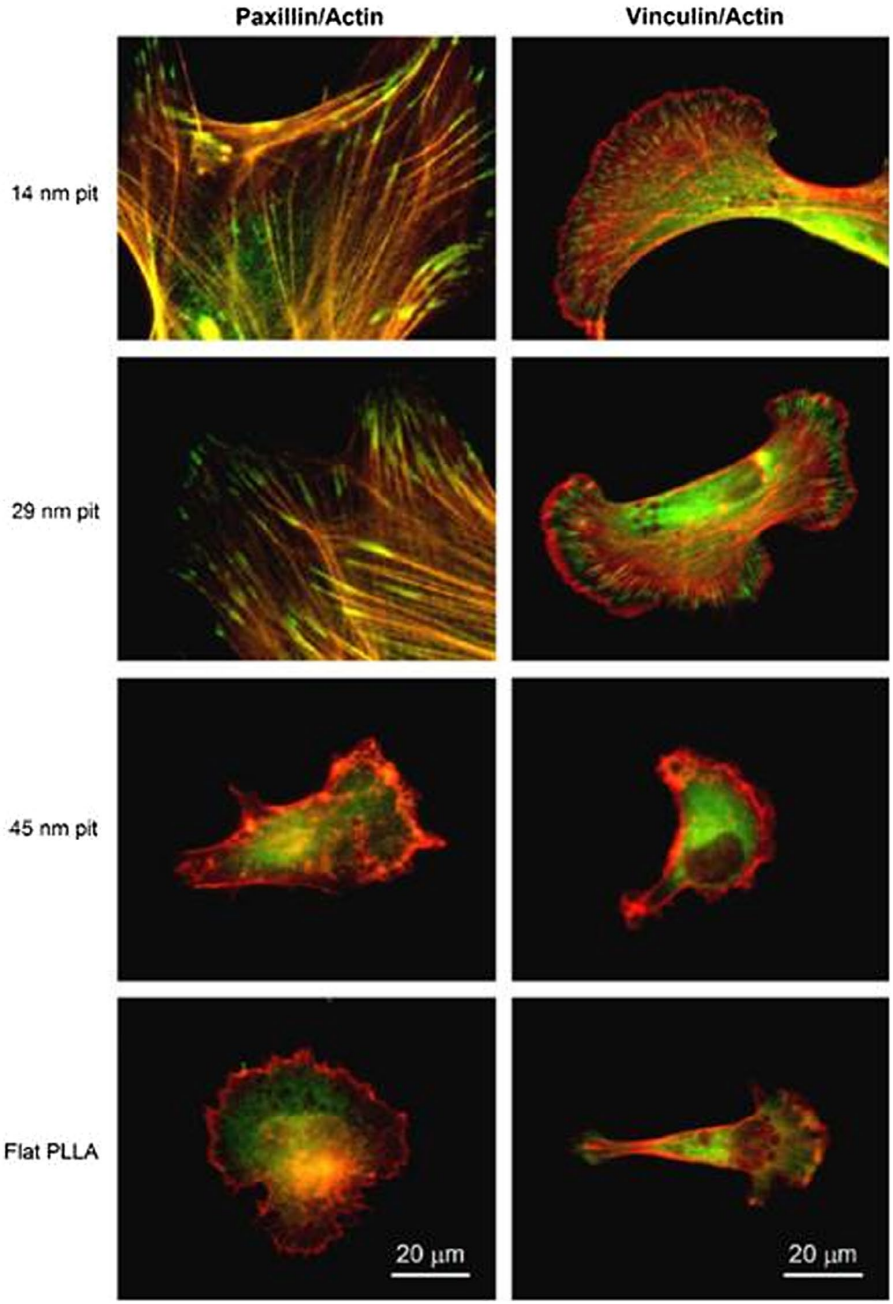
Paxillin (green) and vinculin (green) immunofluorescence staining double-labeled with actin (red) for hFOB cultured for 24 h on PLLA/PS demixed nanopit-textured films and flat PLLA films. Images reproduced with permission from [46].

Generally, cellular mechanotransduction relies on the ability of proteins of the focal adhesion to change chemical activity state when physically distorted and convert the mechanical energy into biochemical energy by modulating the kinetics of protein–protein or protein–ligand interactions within the cell.[45] The mechanism of hFOB response to nanoscale patterns made by polymer demixing is explored in a study by Lim et al. [46] Nanopits of 14 nm and 29 nm in depth (Figure 2), made from PLLA and PS, were observed to induce better cell attachment and adhesion compared 45 nm nanopits and flat PLLA. Paxillin and vinculin expression (Figure 3), as well as Western blotting results, are also consistent with the formation of cell adhesion. To be specific, significantly higher phosphorylated FAK (pY397), FAK and *α*v integrin expression were observed on the shallower nanopits after 24 h of culture. Although little is known about the effects of nanoscale features on integrin-mediated activation of adhesion proteins and downstream signaling pathways, results from this study support the theory of direct mechanotransduction.

The positive influence of nanoscale topography on cellular response is also observed on osteoprogenitor cells cultured on two types of poly(methyl methacrylate) (PMMA) imprints with different dimensions and spacings.[47] In this study, polymer demixed PMMA imprints are referred to as 3:1000 and 3:3000 (i.e. percentage of polymer in solvent: spin speed) while colloidal lithography PMMA imprint is referred to as hemi (i.e. hemispheres). AFM results revealed that 3:1000 imprint was 45 nm in height, 2.2 μm in diameter and had center-to-center distance of 4.3 μm whereas the 3:3000 imprint was 33 nm tall with diameter of 1.7 μm and center-to-center distance of 2.9 μm. The hemispheres had a defined height of 10 ± 1 nm, diameter of 144 ± 11 nm and spacing of 184 ± 24 nm.

**Figure 4.**
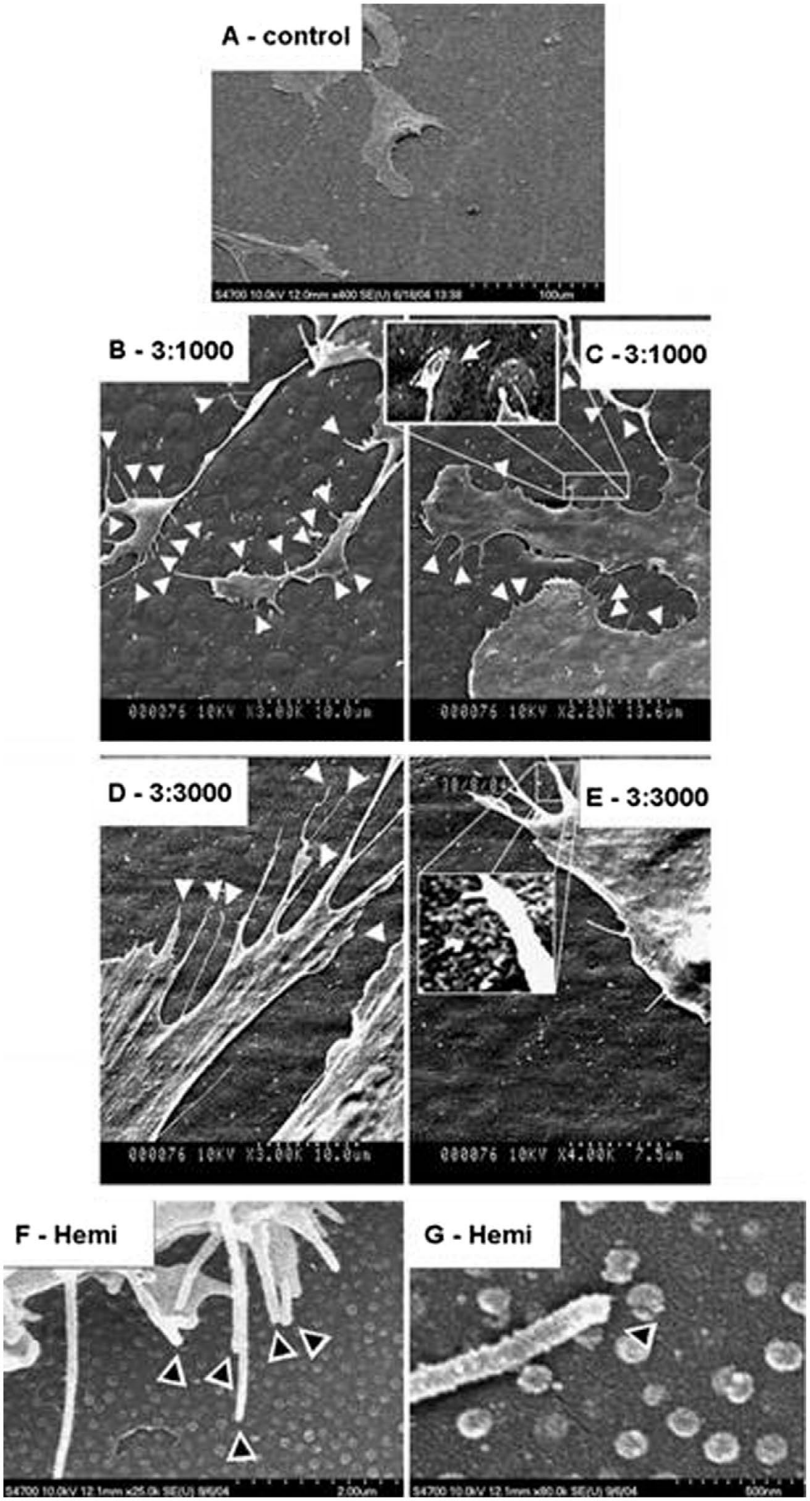
SEM image of (A) human bone marrow stem cells (HMSCs) with normal morphology on planar control materials; (B, C) filopodia interaction with the 3:1000 substrates (arrowheads) and inset on (C) shows filopodia curving around an island; (D, E) filopodia interaction with the 3:3000 substrates (arrowheads) and inset on (E) shows filopodia curving around an island; (F) filopodia interaction with the hemi substrates (arrowheads); and (G) filopodia curving around a hemisphere. Images reproduced with permission from [47].

**Figure 5.**
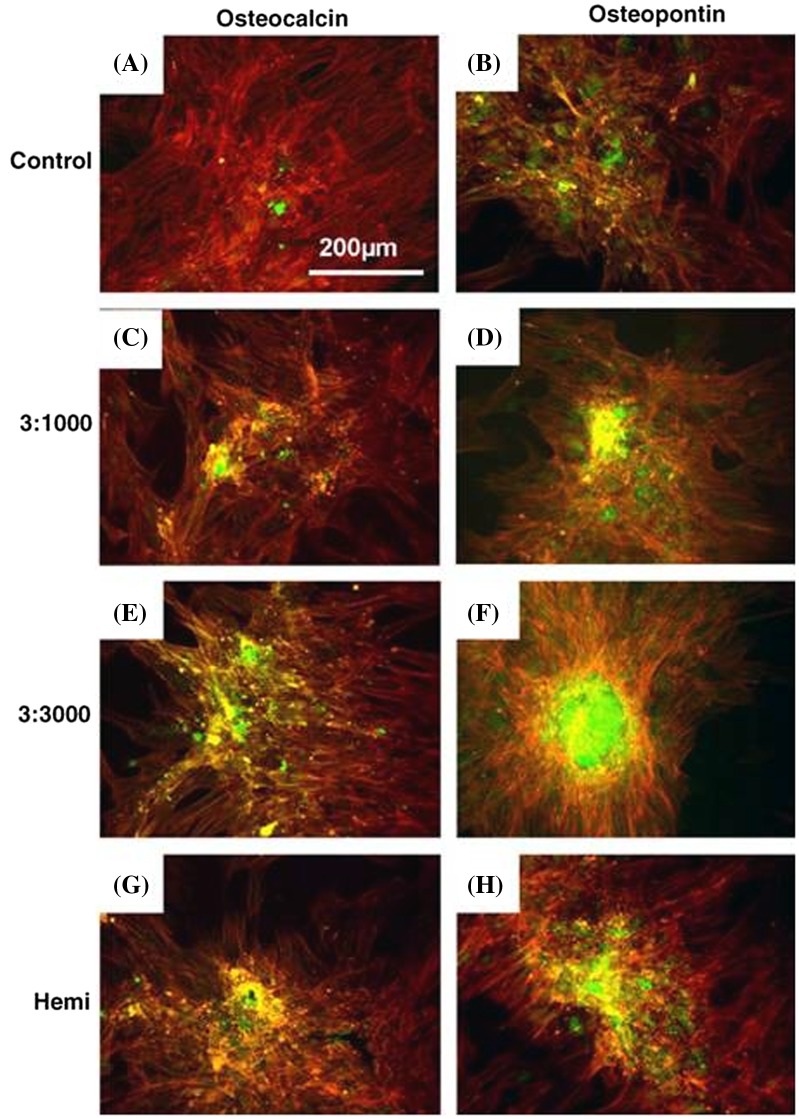
Osteocalcin (OCN) and osteopontin (OPN) fluorescence images of HMSCs cultured on control and test materials. Cells on planar control formed confluent layers but very little OCN or OPN stained was observed on day 21. Bone nodule formation can be seen on 3:1000. (Note: red = actin, green = OCN/OPN). Images reproduced with permission from [47].

The results revealed a significant increase in cell spreading on all the nanotopographies compared to cells cultured on planar control. Cells were found extending filopodia on hemi and curling around 3:1000 and 3:3000 samples (Figure 4). Cells also showed enhanced expression of stress fibers and tubulin as well as vimentin networks on nanotopographies. OCN and OPN expression which indicate the maturation of osteoblast population and subsequent mineralization were found to be higher on nanotopographies compared to control after 21 days of culture (Figure 5). Not only that, bone nodules could be seen on the 3:3000.

It is widely acknowledged that stem cells are affected by ECM properties. Tension caused by size variations and physical deformation within the matrix can change the cell shape and cause cell distortion that often leads to guided cell fate. Soft matrices that mimic the brain have been observed to trigger neurogenic lineage commitment, whereas stiffer matrices mimicking muscle and collagenous bone have been found to cause myogenic and osteogenic differentiation respectively.[48]

Dalby et al. [49] studied the influence of surface structure on lineage-specific differentiation of stem cells.^[49]^ The nanopits (120 nm in diameter and 100 nm depth) were arranged in a square array (SQ) with center-to-center spacing of 300 nm, in a hexagonal array (HEX), a disordered square array with dots displaced randomly by up to 50 nm on both axes from their position in a true square (DSQ50), a disordered square array with dots displaced randomly by up to 20 nm on both axes from their position in a true square (DSQ20) and pits placed randomly over a 150 μm by 150 μm field, repeated to fill a 1 cm^2^ area (RAND).

Two types of cells were seeded on planar PMMA and nanopits – osteoprogenitors and MSC. OPN and OCN expression, which demonstrate maturation of the osteoprogenitors and subsequent mineralization on planar PMMA, were absent despite displaying good cell density. Meanwhile, osteoprogenitors cultured on RAND exhibited a denser cell growth (compared with planar PMMA) and very limited OPN and OCN expression. Dense aggregates similar to bone nodules were observed in osteoprogenitors cultured on DSQ50 along with an increased level of OPN and OCN expression. The MSCs were fibroblastic in appearance with a highly elongated and aligned morphology on planar PMMA and SQ while on RAND they exhibited typical osteoblast morphology with negligible OPN and OCN-positive areas after 21 days.

**Figure 6.**
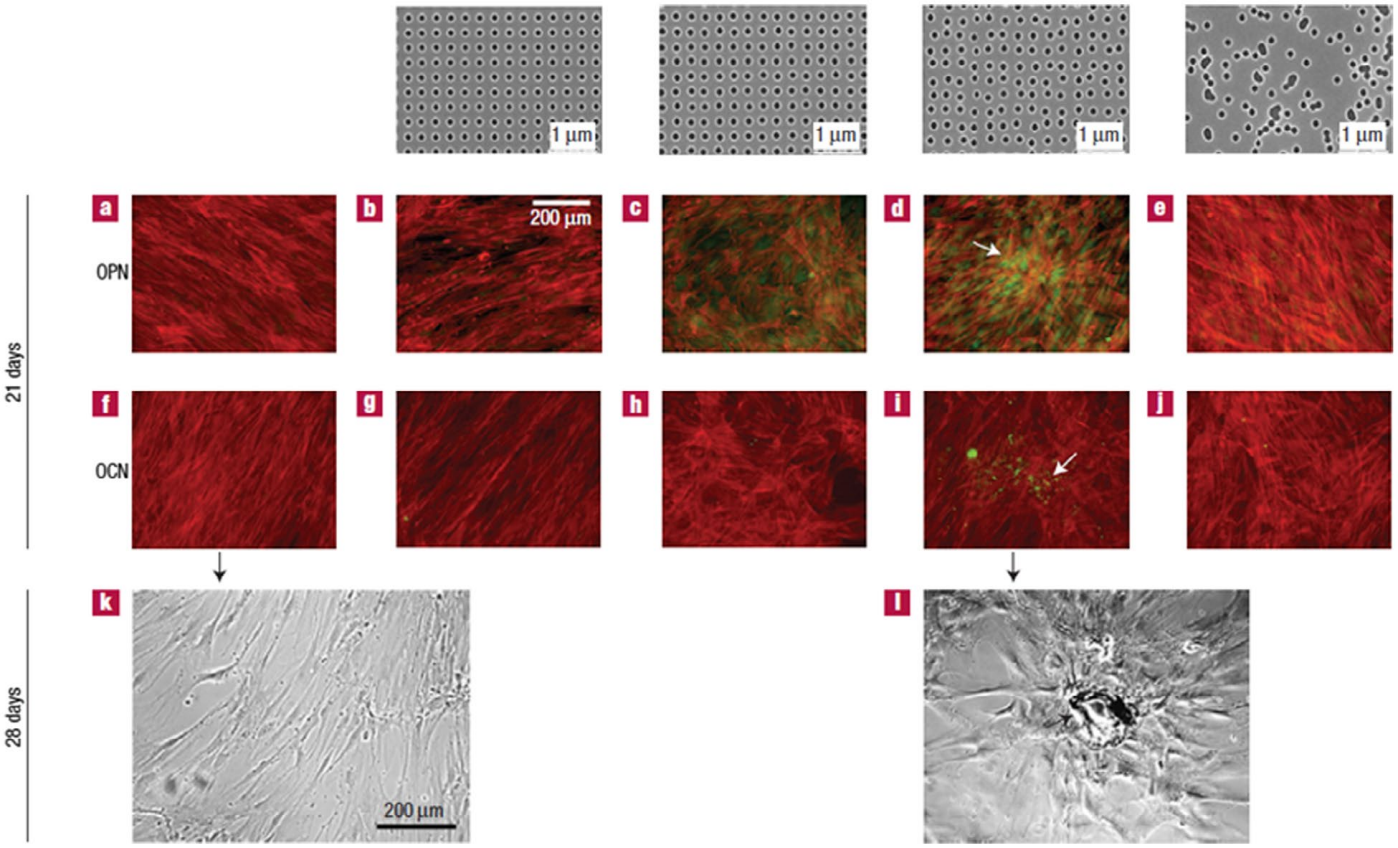
The topmost row shows the images of nanotopographies fabricated by electron beam lithography (EBL). All the pits are 120 nm in diameter, 100 nm deep and have average 300 nm center–center spacing with square, displaced square 20 (±20 nm from true center), displaced square 50 (±50 nm from true center) and random arrangements. (a, f) MSC with fibroblastic appearance and absence of OPN or OCN positive cells on the control, (b, g) no OPN or OCN positive cells on SQ, (c, h) OPN positive cells but no OCN positive cells on DSQ 20, (d, i) OPN and OCN positive cells and nodule formation (arrows) on DSQ 50, (e, j) MSC with osteoblastic morphology and absence of OPN or OCN positive cells on RAND, (k) MSCs with fibroblastic morphology on control after 28 days of culture and (l) mature bone nodules on DSQ 50 after 28 days of culture. Images reproduced with permission from [49].

MSC on DSQ20 (replaces HEX) exhibited similar morphology and OCN expression but expressed foci of OPN. Meanwhile, MSCs cultured on DSQ50 showed discrete areas of intense cell aggregation, early nodule formation, positive OPN and OCN expression and the only that exhibited positive identification of mineralization within the discrete nodules (Figure 6) after 28 days of culture. The results revealed that highly ordered nanotopographies produced low to negligible cell adhesion and osteoblastic differentiation. Cells on random nanotopographies showed a more osteoblastic morphology by Day 14 although with slightly raised OPN and OCN expression.

**Figure 7.**
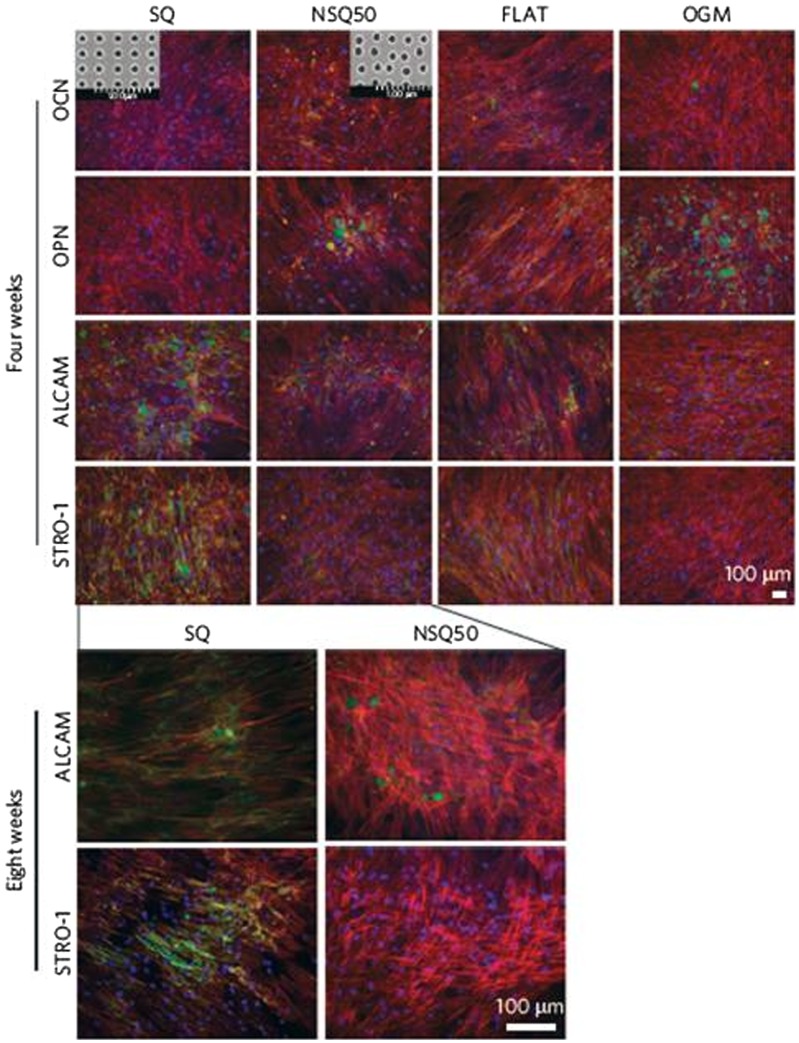
Expression of progenitor and osteoblast markers by MSCs cultured on SQ, NSQ50 and controls (i.e. flat and osteogenic media (OGM)) after four and eight weeks of culture and the insets show SEM images of the SQ and NSQ50 surfaces. (a) On the flat surface the cells had fibroblast-like morphology and the heterogeneous cell population, retained stromal precursor antigen-1 (STRO-1) and activated leukocyte cell adhesion molecule (ALCAM) expressions (i.e. the MSC markers) and expressed OCN and OPN markers. On the OGM control, the expression of the less specific progenitor marker, ALCAM, was retained while OCN and OPN expressions were noted. The cells had grown confluence on SQ and no expression of OCN or OPN was noted. STRO-1 and ALCAM markers were highly expressed on SQ but only low levels of STRO-1 were noted on NSQ50. (b) STRO-1 is a more stringent marker for MSC than ALCAM which is expressed by both stem cells and progenitor cells. Expression of ALCAM on NSQ50 at eight weeks suggests that there are still osteoprogenitor cells present although the actual MSC numbers have dwindled. In all images, green = phenotypic marker, red = actin (cell morphology) and blue = nucleus. Images reproduced with permission from [50].

Quantitative polymerase chain reaction (qPCR) analysis on nanopits with similar diameter, center-to-center spacing and offset (120 nm pits in square arrangement with center-center spacing of 300 nm and ±50 nm offset in both *x*- and *y*- axes), conducted by McMurray et al. [50] also found that MSC differentiate to osteogenic cells on NSQ50, a relatively ‘random’ nanopits but retained MSC markers and multipotency on absolute square lattice symmetry (SQ) nanopits. Figure 7 shows the expression of progenitor and osteoblast markers by MSC on SQ, NSQ50 and controls after four and eight weeks of culture. Both studies [49,50] have shown that MSC fate can be influenced by symmetry and regularity of nanotopographical patterns. Nanodisplaced topography can increase osteospecific differentiation while square lattice symmetry nanopits (SQ) have the potential to retain MSCs markers for prolonged periods. Although the reason behind these phenomena was not explained in the article, there is a chance that local disorder of ECM may have promoted the emergence of integrin activation and focal adhesion formation under a constant global average of ECM ligand density.[51]

**Figure 8.**
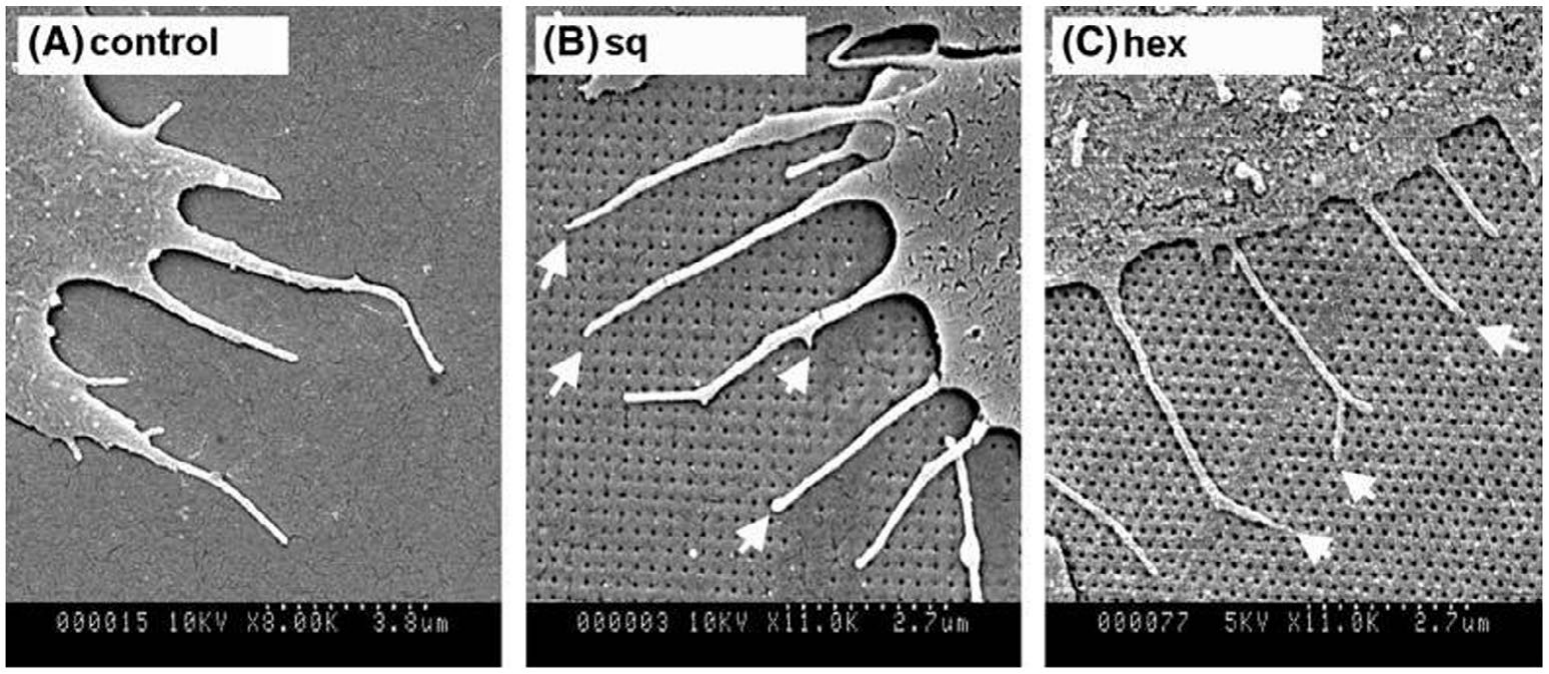
Scanning electron micrographs of filopodia of osteoprogenitor cells cultured on (A) planar control; (B) square (SQ) nanopit arrays and (C) hexagonal (HEX) nanopit arrays. Images reproduced with permission from [52].

In another study, osteoprogenitor cells cultured on polycarbonate (PC) imprints of 120 nm diameter pits with 300 nm center-to-center spacing in square (SQ) and hexagonal (HEX) arrangements were observed to exhibit stellate cell morphology and less spread compared to the planar control.[52] The number of filopodia produced per μm (Figure 8) of membrane in cells and the percentage of filopodia–pit interactions also were higher on the pits compared to planar control. Fluorescence staining confirmed the observation although vinculin results showed that nanotopographies reduced cells ability to form focal adhesion with very few notable adhesions seen on square and hexagonal pits.

Unlike other patterns, grooved nanostructures allow control of cell alignment and morphology by triggering reorganization of the cell cytoskeleton in the direction of the grooves. Basically, they influence the formation of focal adhesions by simultaneously providing vertical ledges which disrupt integrin binding as well as topographically planar areas which facilitate integrin binding.[53]

**Figure 9.**
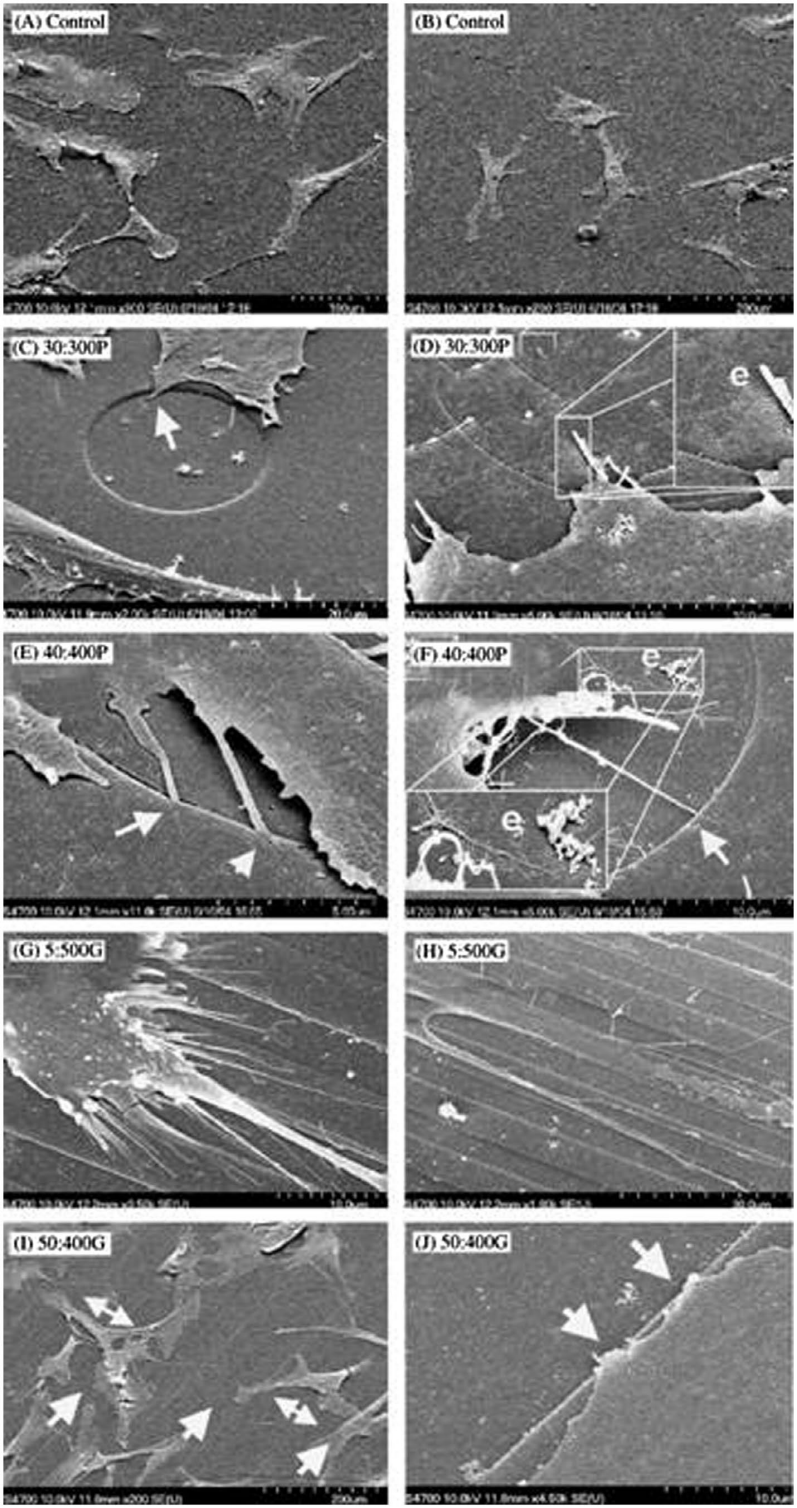
SEM images of (A, B) hBMCs with normal morphologies on planar control materials; (C) hBMC conforming to a groove edge of smaller and shallower pit (arrow); (D) hBMC with filopodia entering a small pit and inset (e) shows evidence of endogenous matrix formation; (E) filopodial guidance in larger and deeper pit (arrow); (F) filopodial guidance (arrow) and inset (e) shows evidence of endogenous matrix formation; (G, H) contact guidance of hBMC and their filopodia on narrow grooves; (I) hBMC aligning along the wide grooves (arrow) and spanning across grooves (double headed arrows); (J) filopodial guidance on the wide grooves (arrows). Images reproduced with permission from [54].

**Figure 10.**
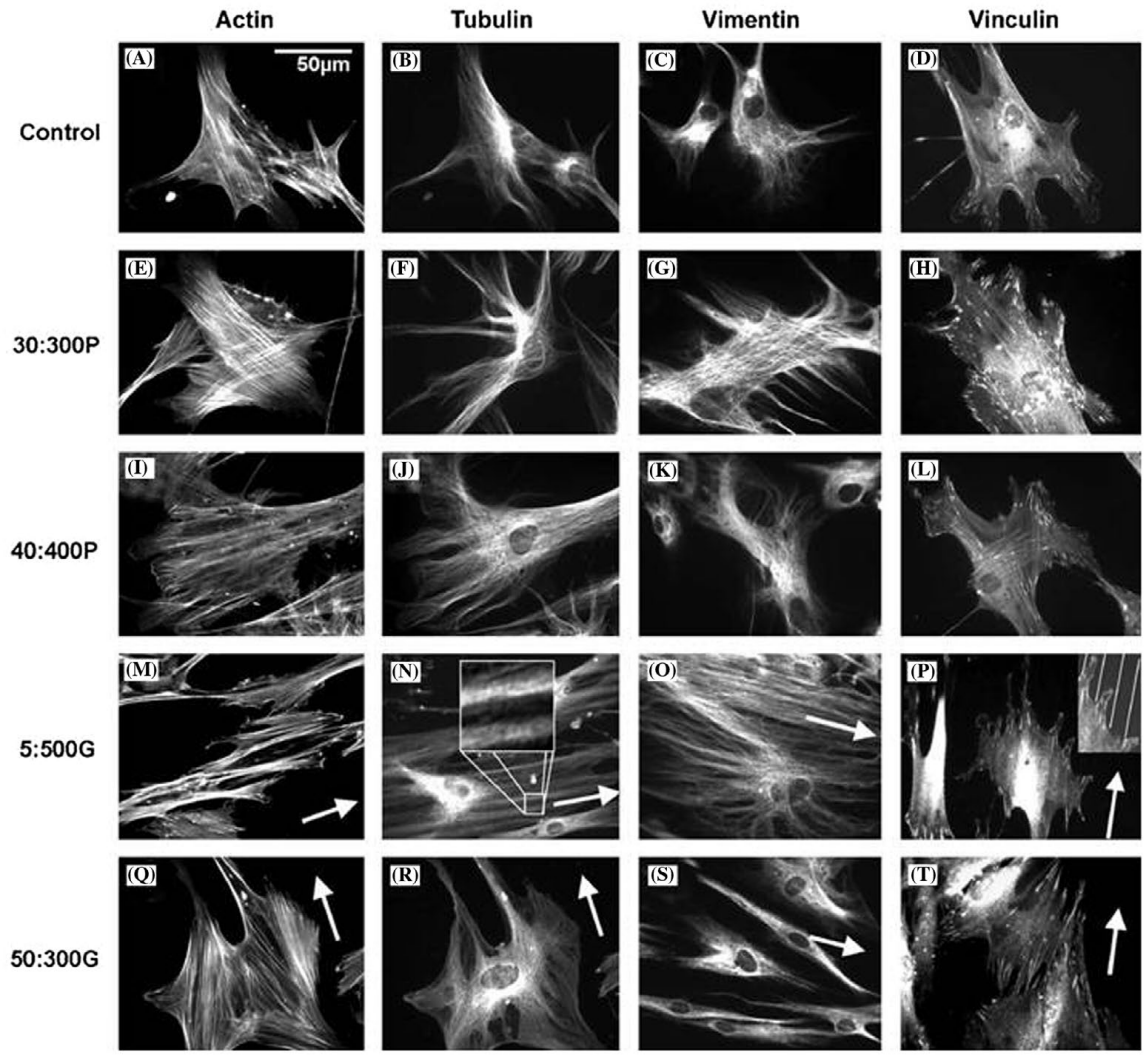
Fluorescence images of actin, tubulin and vimentin cytoskeletons and vinculin (focal adhesions) of HBMSCs cultured on control and test materials. Increased cytoskeleton organizations and numbers of focal adhesions were seen on the topographies compared to cells on planar control. (N) tubulin is seen condensing along grooves while in (P), adhesions are seen aligning to the grooves. Images reproduced with permission from [54].

In a study that compares human bone marrow cells (hBMCs) response to nanopits and nanogrooves, Dalby et al. [54] discovered that hBMCs formed a larger cell area and more defined and organized stress fibers (Figure 9) on larger and deeper pits with diameter:depth ratio of 40:362 nm (denoted as 40:400P) compared to their smaller and shallower counterparts with diameter:depth ratio of 30:310 nm (denoted as 30:300P) and grooves (width:depth ratio of 5:510 nm (denoted as 5:500G) and 50:327 nm (denoted as 50:300G)).^[54]^ However, increased OCN and OPN levels were observed on both pit patterns after 21 days. Cytoskeleton arrangement with highly aligned stress fibers was more pronounced on 5:500G and an image of condensed tubulin and vimentin network on the similar structure can be seen in Figure 10.[54] Increased cell sensing, adhesion, spreading, cytoskeletal organization and OPN and OCN production on these highly ordered topographies strongly suggest that topography could be used to influence osteoprogenitor differentiation to mature, mineral producing osteoblasts.

Nanostructures have been shown to induce significant modulation of focal adhesion formation, cytoskeletal development, and cellular spreading which eventually lead to functional differentiation. In this review, three different types of nanoscale patterns have been discussed: nanoprotrusions, nanopits and nanogrooves. In a review article, Biggs et al. [45] explain that focal adhesion is activated on nanoprotrusion with dimension less than 70 nm and feature spacing between 70 and 300 nm. However, nanoscale features with lateral dimensions >300 nm (provided that feature height/depth <70 nm) was found not to perturb focal adhesion formation either. In fact, it increases the total surface area which enables cells to establish cell–substratum contacts that consequently increases integrin–ligand interactions. On nanopits, while focal adhesion is reinforced on pit diameter and depth <70 nm, increased potential for protein capture in the absence of diminished integrin binding was observed when the inter-feature spacing is >70 nm. As for the nanogrooves, ridge structure <70 nm and groove widths between 70 and 300 nm were found to disrupt focal adhesion formation. ^[45]^

Both hFOB and MSC are overtly responsive to nanoscale topography and their primary response to the nanopatterns are pretty similar. Initially, they would form focal contact with the surface of the material but subsequent responses are very much dependent on the dimensions and arrangements of the nanopatterns.

The signaling mechanism of bone synthesis within terminally differentiated osteoblasts includes early electrophysiological responses followed by activation of intracellular signaling pathways. These mechanisms then regulate the actions and transcription levels of transcription factors involved in the regulation of the osteoblast phenotype hence regulating the genes that code for bone matrix proteins.[55]

Normally, ALP is expressed during the matrix maturation phase whereas OCN, bone sialoprotein (BSP), and OPN are expressed at the beginning of the matrix mineralization phase. Matrix proteins such as OCN is one of the few osteoblast specific genes that is abundantly present in the bone.[56] It plays an important role in the differentiation of osteoblast progenitor cells as demonstrated by significant up-regulation in matrix synthesis and mineralization.[57] However, unlike OCN, ALP and OPN are not bone specific but they act as an early indicator of cellular activity and differentiation, and are involved in cell adhesion, migration and survival respectively. Like other osteoblastic genes, BSP is regulated by both chemical and physical cues and plays a role in the early mineralization of osteoblasts.[58]

Bone morphogenetic proteins (BMPs), in particular BMP-2 and BMP-6, have been found to strongly promote osteogenesis in MSCs.[59,60] BMP-2 induces the p300-mediated acetylation of Runt-related transcription factor 2 (Runx2), a master osteogenic gene which leads to enhanced Runx2 transactivating capability. In this review, levels of OCN and OPN have been shown to significantly upregulated in response to nanoprotrusions,[47] disordered square array (DSQ50) of nanopits,[49] and both nanopits and nanogrooves.[54]

Leukemia inhibitory factor (LIF),[61,62] fibroblast growth factors (FGFs),[63,64] and mammalian homologs of *Drosophila* wingless (Wnts),[65,66] are among the genes involved in MSC stemness maintenance. Square lattice symmetry nanopits (SQ) [50] in this review have been observed to retain MSC characteristics but the expression of the aforementioned self-renewal genes was not analyzed. Instead, the lack of expression of OPN and OCN was used as an indicator for MSC stemness. It is worth noting that the genes mentioned here form only part of the complex regulatory systems involved in osteoblast and MSC differentiation.

The objectives of the studies discussed in this review revolve around the same theme which is to compare cellular response between nanostructures that are fabricated with slight differences in *x*, *y*, and *z* dimensions and to compare these structures with a planar structure that is structurally comparable to TCPS. It is evident from the studies that slight differences in dimension and arrangement of the nanostructure have a significant effect on the way cells respond. Although there are many aspects at play and no one has yet cracked the complex mechanism of cellular response, topography is one of the surface characteristics that can be easily manipulated to modulate cellular activity.

Nanotopographies have been envisioned to be used as platforms for culture of large quantities of high-quality stem cells, yet access to hi-tech equipment to make uniform and high resolution pattern is very limited in many places. Indeed, there is a trade-off when one chooses a technique over the other, but for biological investigations, nanofabrication techniques that can make reproducible patterns across a large surface area are of the utmost importance. Table 1 summarizes hFOB and MSCs response to nanotopographies.

**Table 1.** Summary of cell response to nanotopographies.

Technique	Materials used	Pattern and dimension	Cell type	Cell responses	Ref.
Polymer demixing	PS/PBrS	Nanoislands with an average height of 11, 38 and 85 nm respectively	hFOB	Higher cell adhesion and larger cell size on 11 nm nanoislands.	[42]
				Similar ALP activity on 85 nm nanoislands and TCPS	
Polymer demixing	PMMA	**3:1000 imprint** (height:45 nm, diameter: 2.2 μm, center-to-center distance: 4.3 μm)	HMSCs	Increased cell spreading, enhanced expression of stress fibers, tubulin and vimentin networks and higher OCN and OPN expression on nanotopographies compared to control	[47]
		**3:3000 imprint** (height: 33 nm, diameter: 1.7 μm, center-to-center distance: 2.9 μm)		Cells extending filopodia on hemispheres and curling around imprints	
		**Hemispheres** (height:10 ± 1 nm, diameter: 144 ± 11 nm, spacing:184 ± 24 nm)			
Polymer demixing	PLLA/PS	Pit-shaped topography of 14 nm, 29 nm, 45 nm deep respectively	hFOB	Higher cell coverage, *α*v integrin and paxillin expression on 14 nm and 29 nm	[46]
				Greatest cell attachment on 14 nm followed by 29 nm and 45 nm pits	
				Invariant vinculin expression on pits	
Photolithography	PMMA	**Pit diameter to depth ratio:** 30:310 nm (denoted as 30:300P) 40:362 nm (denoted as 40:400P)	hBMCs	hBMCs conformed, formed filopodial contact and exhibited increased cell spreading, increased tubulin and vimentin networks as well as OCN and OPN expressions on the pit patterns	[54]
				Larger hBMCs cell area, more defined stress fibers and mature nodule formation (after 21 days) on 40:400P	
		**Groove width to depth ratio:** 5:510 nm (denoted as 5:500G) 50:327 nm (denoted as 50:300G)		Better contact guidance, significant reduction of cell area, highly aligned stress fibers along the groove direction and tubulin condensing along the ridges on 5:500G	
				Aligned vimentin and increased areas of OCN and OPN production on groove patterns	
EBL	PMMA	100 nm deep (D: 120 nm) PMMA imprints arranged:	MSC	Elongated, and aligned morphology with fibroblastic appearance on SQ and planar control	[49]
		SQ: in square array with center-to-center spacing of 300 nm		Decreased osteoprogenitor density on HEX	
		HEX: hexagonal array		Increased level of OPN, OCN and mineralization on DSQ50 after 28 days	
		DSQ50: randomly up to 50 nm on both axes from their position in a true square		Osteoblastic morphology and expressed foci of OPN on DSQ20	
		DSQ20: randomly up to 20 nm on both axes from their position in a true square		Denser cell growth on RAND compared with planar PMMA	
		RAND: randomly over a 150 μm by 150 μm field, repeated to fill a 1 cm^2^ area		Polygonal, osteoblastic morphology on RAND after 21 days	
EBL	PC	PC imprints comprised of 120 nm diameter pits with 300 nm center-to-center spacing in SQ and HEX arrangements	Osteoprogenitor cell	Stellate cell structure, higher number of filopodia per μm of membrane and presence of cortical actin on PC imprints	[52]
				Higher cell spread on hexagonal arrangement and planar control Stress fibers on planar control	
EBL	Polycaprolactone (PCL)	SQ: PCL imprints comprised of 120 nm deep pits in square arrangement with center-center spacing of 300 nm	MSC	SQ induced a switch from osteogenic stimulation to a surface conducive to MSC growth and permitted prolonged retention to MSC markers and multipotency	[50]
		NSQ50: PCL imprints comprised of 120 nm deep pits with ±50 nm offset in both x- and y- axes		MSC differentiated into osteogenic cells on NSQ50 and OGM controls after four and eight weeks	
				STRO-1, ALCAM, OPN and OCN markers expressed by cells on NSQ50 and OGM	
				Increased OPN expression on NSQ50 and OGM after a few weeks	
				Cells exhibited raised/similar metabolomic profiles on NSQ50 and OGM compared to SQ	

Abbreviation: OGM, osteogenic media.

## 3. Emerging nanofabrication technique and future direction

Nature has demonstrated its ability to produce complex living organisms by self-organization. The limitations of top-down nanofabrication techniques including low throughput and high production cost may be solved by taking cues from nature. Nanoscale structures that can self-organize into a specific formation or pattern may bypass the complex and expensive lithographic methods. Additionally, if the process can be controlled to make patterns ‘to order’, it will bring the nanofabrication technology to a whole new level.

The idea of self-assembly is not new. In fact, such attempts have begun almost two decades ago by chemists and biologists.[67] The self-assembly approach involves manipulation of molecules by the interaction of molecular forces and other inter-particle forces. Carbon nanotubes (CNTs) and self-assembled monolayers (SAMs) are some of the examples of self-assembled nanostructures which can be found in applications such as nanoscale patterning.[68]

The self-assembly technique offers a simple and low-cost approach to make large-area periodic nanostructures. However, self-assembly as a stand-alone method for nanofabrication is presently unable to produce structures with precise spatial positioning and arbitrary shapes.[69] Not only that, it also presents higher density of defects compared to conventional nanofabrication techniques. In this case, templated self-assembly which uses top-down lithographic approaches to provide the topographical and/or chemical template to guide the bottom-up assembly of colloidal particles and macromolecules, may bring out the best of the two approaches.[70]

**Figure 11.**
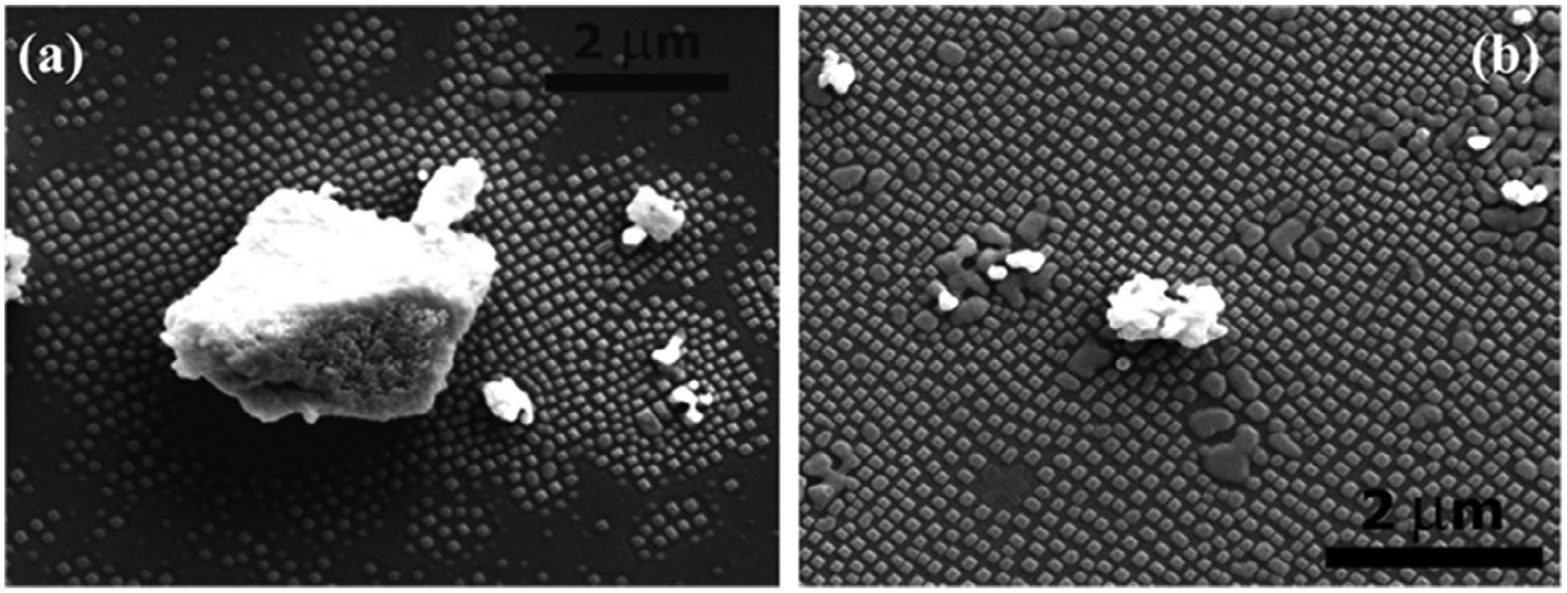
SEM micrograph of (a) nanoislands around a gadolinia-doped ceria (GDC) particle after annealing at 1100 °C for 5 h with 10 °C min^–1^ heating and 1 °C min^–1^ cooling rates; and (b) smaller powder particles with relatively broader nanoisland coverage after heat treatment. Images reproduced with permission from [71].

There is an emerging technique that exploits the intrinsic chemical properties of rare earth metal oxide powder to make self-assembled patterns on single crystal substrates. An array of island-like patterns was found by accident by M.D. Rauscher after he annealed a (100)-oriented yttria-stabilized zirconia (YSZ) substrate coated with GDC.[71] Rausher’s idea was further explored by H. Ansari who took a different approach to distribute the GDC powder on the same substrates. After he managed to get nanoislands between GDC patches prepared by photolithography, he prepared a low loading powder suspension (i.e. GDC in deionized water) and deposited a small amount of the powder suspension onto YSZ-(100) substrate before annealing the sample at a high temperature. SEM results revealed that nanoislands formed around GDC powder particles on YSZ-(100) (Figure 11). According to Ansari, the patterns formed as a result of strain induced by lattice mismatch between the doped thin layer and the YSZ substrate during soak time, which later break up into self-assembled nanostructures to relieve the strain. The alignment is controlled by the elastic modulus anisotropy of the substrate.[72]

**Figure 12.**
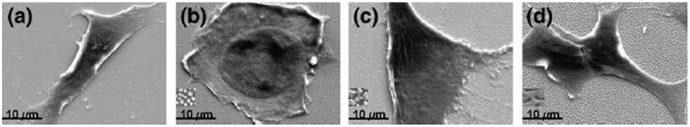
SEM of morphology of representative SK-N-SH neuroblastoma cells on the (a) smooth control; (b) islands; (c) connected islands, and (d) pits. Images reproduced with permission from [74].

The patterns produced by this technique have been transferred to polymeric materials [73] and used in a study of the effect of nanotopographies on neuron cell attachment (Figure 12).[74] The nanopatterns of GDC-YSZ have the potential to be used as molds for pattern transfer to polymeric materials of biological interest such as gelatin,[75] poly(ethylene glycol) dimethacrylate (PEG-DMA),[76] poly-methylmetaacrylate (PMMA),[77] PS,[78] or poly-dimethylsiloxane (PDMS),[79] before being used as a platform for cell biology research. The powder method holds a tremendous future in the nanofabrication industry and therefore a systematic approach to control the size, shape and orientation of the patterns has to be achieved to ensure a reproducible outcome. Research on technique optimization is ongoing and can be expected in the next publication from this group.

## 4. Conclusions

In this review, the influence of nanoscale topographies on osteoblast and MSC response has been discussed. Although materials such as PMMA, PLLA, PS, PC and PCL provide tools for probing cell adhesion, mechanics, and functions, it is difficult to reach a consensus on the effect of nanotopography on cellular response, due to differences in the nanofabrication techniques, topographical dimensions and types of cells. Advances in high-resolution nanofabrication techniques have made it possible to establish cellular response guidelines but there is an urgent need for easy, fast and scalable patterning techniques in regenerative medicine. Self-assembly techniques such as the powder method offer an alternative way to fabricate nanoscale pattern with controlled nanodisorder. The introduction of facile materials engineering approach in regenerative medicine may promote efficient cell expansion or lineage-specific differentiation as well as significantly reducing cell bioprocessing expenses.

## Funding

This work was supported by the University of Malaya High Impact Research (HIR) [grant UM.C/HIR/MOHE/ENG/44]; Institute of Research Management & Monitoring (IPPP) [grant PG117-2014A]; and University Malaya Research Grant [grant RP040A-15HTM].

## Disclosure statement

No potential conflict of interest was reported by the authors.
